# Perceptions and Experiences of HIV Nurses on Peer Support for People Living With HIV in the Netherlands: A Qualitative Study

**DOI:** 10.1177/23259582251372442

**Published:** 2025-09-04

**Authors:** Maarten Bedert, Kevin Moody, John de Wit, Pythia Nieuwkerk, Marc van der Valk

**Affiliations:** 1 Division of Infectious Diseases, 26066 Amsterdam Infection and Immunity Institute, Amsterdam University Medical Centers, University of Amsterdam, Amsterdam, the Netherlands; 2 Department of Interdisciplinary Social Science, 8125 Utrecht University, Utrecht, the Netherlands; 3 Department of Medical Psychology, 26066 Amsterdam Public Health Research Institute, Amsterdam University Medical Centers, location AMC, University of Amsterdam, Amsterdam, the Netherlands; 4 198321 HIV Monitoring Foundation, Amsterdam, the Netherlands

**Keywords:** HIV care, peer support, HIV nurses, chronic care, integrated care, informal care

## Abstract

**Background:**

Peer support is an important intervention to achieve increased quality of life for people with HIV. We set out to understand the perceptions and experiences of HIV nurses with peer support in the Netherlands.

**Methods:**

We conducted 21 semi-structured interviews which were analysed using thematic analysis.

**Results:**

Regarding referral to peer support services, we found that nurses favoured patients with the larger presumed burden of living with HIV. Nurses identified logistical and personal barriers with referrals: additional workload, lack of belief in peer support programs, concerns about the impact on their patients because of earlier unfavourable experiences with peer support. Patients are often considered not being ready for peer support and are fearful of unwanted disclosure by others. A good personal connection with peers and having peers in active care facilitated linkage to peer support by increasing visibility and proximity.

**Conclusions:**

We suggest that closer integration of peer support into formal care is a possible solution to the existing barriers.

## Introduction

Recent biomedical developments suggest that living with HIV is considered a manageable, chronic condition.^1^ Life expectancy of people living with HIV (PLHIV) is similar to that of people without HIV, and people on treatment have a viral load that becomes undetectable and therefore cannot be transmitted to others (undetectable = untransmittable; U = U).^2^ As a result, HIV care is increasingly structured following chronic care models (CCM), whereby more attention is given to patient-centred care and quality of life.^3^ The CCM emphasises self-management and involvement of people with chronic diseases, which has shown to improve clinical outcomes for PLHIV.^4,5^ However, despite clinical advances, many PLHIV continue to experience significant disease burden due to the presence of comorbidities, and a higher likelihood of depression, social isolation, stigma and discrimination.^6-9^ Peer support is a tool to encourage better HIV management among PLHIV and to reduce the experience of stigma.^10^ Facilitating peer support programmes also increases the involvement of PLHIV in the continuum of HIV care.^11^ Peer support is defined as ‘the giving of assistance and encouragement by an individual considered equal’.^12^^(p.323)^ Whereas non-disclosure of HIV status could inhibit support, peers can offer patients guidance on issues affecting them, such as stigma, beyond the confines of formal care, which may address the quality of life of PLHIV. Even though definitive data are hard to come by, involvement of peers in care likely also has positive effects on HIV prevention, retention in care, and on treatment adherence, which is crucial for effective HIV self-management and efforts to curtail the epidemic.^13-15^

In the Netherlands, about 21,500 people are currently in routine clinical care in one of 24 specialised HIV treatment centres regulated and approved by the national government.^16^ Of all PLHIV in the Netherlands, 94% are currently in care and 96% of those have an undetectable viral load.^16^ Of all PLHIV in the Netherlands, 63% are men who have sex with men (MSM), which is also the main transmission route, and 42% have a migration background, with 13% of those from Sub-Saharan African heritage.^16^ The large majority of PLHIV, among them many people with a migration background, are in care in larger cities like Amsterdam, Rotterdam or The Hague, all located in the west of the country.^16^ In general, PLHIV who have an undetectable HIV-1 viral load visit the treatment centre every six months.^17^ Treatment is provided by a medical specialist and an HIV nurse. Informal HIV care, outside of the clinic, relies for a large part on self-care and support from family members, community organisations and non-governmental organisations (NGOs).^18,19^ Peer support is organised by a variety of national and local NGOs and community organisations. Three peer support programs operate in the context of a recently established Dutch National HIV Alliance. The *HIV Vereniging*, the national patient interest organisation, is based in Amsterdam and provides peer support nationwide through their peer counselling program. They mainly offer one-on-one, short-term peer support and organise a workshop series led by peers. Shiva foundation, also based in Amsterdam, operates separate peer counselling programs for women and men nationwide. For the most part they provide peer support services to PLHIV of African or Caribbean heritage. Third, Mara foundation operates in Rotterdam and the Hague and offers broad social support to a diversity of specific population groups, and also offers one-on-one peer support for PLHIV.

*HIV Vereniging* reports that little over half of their peer support clients are referred by health care professionals, while for Shiva and Mara the majority of their clients are. Following an inquiry, all three organisations report that for 2022, about 150 PLHIV were registered for individual peer support, while not all of these were matched with a peer.

Most PLHIV are introduced and referred to peer support by HIV nurses who play a crucial role in linking formal and informal care.^16^ This raises questions about the nurses’ relationship with their patients as well as peer support providers, and their motives for (non)referral. Given that the organisation and implementation of peer support is largely dependent on the organisation of care in specific settings,^20-22^ this study fills an important gap in knowledge by giving voice to the health care professionals who facilitate peer support. This research project sets out to understand the experiences with and perceptions of peer support among HIV nurses throughout the Netherlands to provide new insights that can contribute to further improving peer support projects and optimising the linkage between formal and informal HIV care.

## Materials and Methods

### Study Design and Procedures

Given that our study aimed to describe HIV nurses’ experiences with peer support, this study was designed as a qualitative, phenomenological research project using semi-structured, in-depth interviews. An interview guide was developed based on literature research, informal conversations with nurses, and participant observation at the HIV clinic of the Amsterdam University Medical Centres (UMC). We developed open-ended questions that allowed reflection on the lived experiences of nurses and facilitated bracketing (ie, avoiding the researcher's own preconceptions and biases), both of which are principles of phenomenological design. The Interview Guide was co-created by the team of authors, consisting of qualitative researchers, a policy expert, medical specialists and an activist from the community. This initial interview guide was further developed and refined based on four preliminary interviews with HIV nurses working in different clinics, which were not audio-recorded but for which detailed notes were taken. These initial interviews do not feature in the results section of this study.

### Participants and Recruitment

All HIV nurses working in any of the 24 HIV treatment centres were eligible for participation in this study. Participation was voluntary and based on informed consent. Participants were informed that they could withdraw at any time without consequences. We aimed to include at least one nurse from each HIV-treatment centre to account for differences in the size of treatment centres, differences between urban and more rural treatment centres and differences in the patient populations and staff composition.

Purposive sampling techniques were used to recruit participants. Participant recruitment was conducted between November 2021 and March 2022. Potential participants were informed about the study through the professional association of HIV nurses. Initial invitation emails were sent to treatment teams at all HIV clinics in the Netherlands. Subsequently, appointments were made with individual nurses who expressed interest in participating. All potential participants received detailed information about the study and provided consent before participation in the study. Interviews were conducted by MB who holds a PhD in social anthropology with prior research experience on HIV late presentation and HIV nursing. Inclusion of participants continued until thematic saturation was reached. To confirm data saturation, two additional interviews were conducted after thematic repetition was observed, and no new themes emerged during these interviews.

### Data Analysis

All interviews were audio-recorded and transcribed verbatim. Thematic data analysis was undertaken using an inductive approach based on open coding, followed by code clustering and reviewing,^23^ supported by MaxQDA software. Data analysis was initiated while interviews were still being conducted. The first author initially coded all interviews and subsequently discussed the outcomes with members of the interdisciplinary research group. The group consisted of a social anthropologist, a medical specialist in internal medicine, a clinical psychologist and an implementation scientist specialising in quality of care. Additionally, peer debriefing was conducted with two HIV nurses, who were not included in this study as participants. Further feedback was gathered during a workshop on peer support for health professionals and peer organisations where preliminary results of the study were presented.

### Ethics Approval

The protocol for this study was considered by the Medical Ethical Committee of the Amsterdam UMC. It was granted an exemption from further assessment because the Medical Research Involving Human Subjects Act (WMO) of the Netherlands does not apply (reference: W21_428 # 21.476).

## Results

### Sample Characteristics

Between November 2021 and February 2022, we conducted 21 interviews with HIV nurses (over a quarter of the total number of HIV nurses in the Netherlands) from 21 of the 24 treatment centres. We conducted interviews over the telephone (*n* = 17), online via Teams (*n* = 2), or in person (*n* = 2). All interviews were conducted during working hours. Interviews lasted on average 45 minutes (range: 28–59 minutes). Participants had an average working experience at the HIV treatment centre of 15 years (range: 3–30 years). Sixteen participants were female (76%) and five were male (23%). Of all participants, 10 were HIV consultant nurses (a general nursing degree), while 11 were specialist nurses (an advanced nursing degree). Two worked in small treatment centres (≤ 400 PLHIV in care), 2 in medium size centres (400–700 PLHIV in care) and 17 in large size centres (≥ 700 PLHIV in care).

### Findings

Seven themes emerged from the interviews. These include the *main characteristics* of peer support as identified by the nurses, the *target population* that would benefit most from peer support according to the nurses, *nurses’ experience of the referral process* to peer support, the *barriers* regarding referral to peer support nurses experience or that they think their patients face, the *facilitators* that nurses perceive to enhance referral to peer support, the *effects of peer support* to reflecting patient benefits, and efforts to *follow up* during subsequent consults.

#### Main Characteristics of Peer Support

In describing the main characteristics of peer support, nurses explicitly made a distinction with their own tasks as professionals. They considered peer support a ‘*kind of care, but more socio-emotional, not medical*’ (interview 11, position 71), thereby explicitly emphasising that ‘*the profession should remain with the professionals*’ (interview 11, position 71). This is an indication that a tension may exists between the boundaries distinguishing formal from informal care. Even HIV nurses living with HIV themselves made the explicit distinction between their role as a nurse and as a peer and were mindful of this distinction: ‘*I occasionally use my own HIV as an example but I have to be careful about that, it shouldn’t become peer counselling in the consultation room*’ (interview 1, position 26). An alternative description paints the picture of a peer as ‘*a friend*’ (interview 3, position 35), emphasising that peers provide support beyond HIV and beyond the clinic. Nevertheless, nurses expect a level of expertise and indicate that peers should be formally trained ‘*on how to coach people*’ (interview 8, position 40). The most often mentioned characteristic of peer support, however, is the importance of sameness in the interaction: ‘*they have their own life experience and that can work in positive ways*’ (Interview 6, position 2).

#### Target Population for Peer Support

Among participants there was a significant gender preference when it comes to identifying the main target group for peer support among their patient population, closely linked to PLHIV's presumed acceptance of their diagnosis. Nurses connected this individual health concern to continued public health efforts in HIV care: ‘*we have a group of patients that we have to keep pushing to come to their appointments or we fear losing them from care […] people who have a hard time showing up risk spreading the virus further*’ (interview 3, position 57-63). Participants identified mainly women and, specifically, women with a migration background as the group that would benefit most from peer support: ‘*the people, for us, that need peer support most, are mostly migrant women with HIV*’ (interview 2, position 1). Regarding these patients, nurses identified more issues with disclosure, stigma, discrimination, social isolation and loneliness because of their migration background. In contrast, nurses indicated that MSM are much less in need of peer support since, as one nurse put it, ‘*my experience is that MSM find each other and if they cannot do that, they are able to locate existing services, like a workshop on the internet or whatever*’ (interview 19, position 7). MSM were considered to have access to peers living with HIV in their own network. Nurses also thought that the social history of HIV, especially its intimate interwovenness with gay sexuality, provides privileges to MSM in the context of HIV care.

#### Experiences With Referrals to Peer Support

Nurses experienced the referral process as requiring customisation based on a patient's needs but also because they experienced continuous changes in their own personal relationship with their patients. There are several specific points in the care trajectory were peer support can be mentioned or discussed with patients and usually linkage to peer support is a continuous process that starts from the first consultation: ‘*we bring it up in the first consultation, like yes, there is peer support, maybe you are not ready now but we bring the topic back regularly*’ (interview 18, position 29). When asked to describe the referral process, nurses described many individual cases of patients to illustrate the personal nature of care trajectories and to highlight the continuous re-evaluation of that care trajectory as needs change over time.

Because of the significance of the relationship with the patients, nurses experienced the referral process as labour intensive and time consuming, both personally and logistically. One nurse commented: ‘*it takes time, lots of conversations, you really have to gain the patient's trust*’ (interview 2, position 1). Recognising the possible barriers for patients, nurses indicated they adopted an active role: ‘*I probably make myself more important than I am, but a lot of patients find [a first encounter] stressful, and I am the familiar face*’ (interview 7, position 46). Nurses also experience logistic challenges as the referral systems are not unified across the different organisations offering peer support. For one of the organisations, one nurse described: ‘*you have to contact the patient, contact the [peer], arrange the meeting, reserve a room, then it is a question whether they will both show up. It happens that the patient, and that happens more often than we think, the patient is too afraid and cancels last minute or doesn’t show up. It is a lot of work, that might be set up differently*’ (interview 6, position 80). Still, nurses feel they can do this because the numbers are low: ‘*because it is not a weekly thing. If it would be weekly, then it would be difficult, but no, not at this moment*’ (interview 13, Position 112). Ideally nurses would want patients to initiate peer support themselves recognizing that they may encounter several barriers.

#### Barriers in Referring to Peer Support

Nurses identified barriers, both logistical and personal, that they experienced in referring patients to peer support. Nurses noted limitations and restrictions in the services provided by the organisations offering peer support. They felt that different organisations favoured specific target populations so that they did not know, for instance, who to refer to for what type of patient ‘*[one organization] seems to be more oriented towards men, yes how to put it, men feel at home there but women less so, that is what I hear from women*’ (interview 2, position 47). Especially nurses operating outside of major urban centres noted a disconnect between the needs of their patients and the initiatives on offer. They felt that peers from the city would not be able to empathise with the lived experience of rural based patients: ‘*My MSM here are not the MSM of the city, there are significant differences there*’ (interview 11, position 133). Lack of time was identified as another important barrier: with increasingly busy consultations, peer support was often simply not on the nurses’ minds. For one nurse, this interview served as a reminder: ‘*we have so much to discuss that [peer support] disappears while it is really important […], you know what, I will put up a poster for myself*’ (interview 15, position 77).

On a more personal level, nurses expressed concern about the quality of the peer programs and a need to protect their patients from sub-standard care. They articulated a lack of trust in the content of the peer programs and the capabilities of peers: ‘*I have the feeling that the peer often reasons from their own situation, like “I did this and now it goes well, if you do the same, it will get better for you as well”. But it is not a copy, it is a different person*’ (interview 16, position 38). Others considered the problems patients face too intense to put onto a peer: “*this is the world upside down*” (interview 14, position 84), one nurse commented. In other words, they felt that the contact with a peer might put pressure on both the patient and the peer.

Nurses also identified potential barriers for patients from their experiences during consultations. An important concern was patients’ possible fear of their HIV status being outed to others through the referral process: “*you need to open up and contact people you don’t know. For some that is really: “I really don’t want anybody with my own cultural background”*’ (interview 19, position 67). In addition, people who had bad experiences in the past are said to close down the option of peer support completely: ‘*[they] don’t know my patient. I recently had a Polish woman who wanted a peer, they didn’t have anybody who spoke the language so they connected her with a man. From the moment I saw that, I thought, this will be uncomfortable, I left and let them get acquainted but that was not a good match. I thought, what a pity, if only they would have considered a different woman*’ (interview 3, position 31). Another important concern was that patients may not be ready for peer contact, either because they are not willing to disclose their HIV status, because there are too many steps and people involved, or because peer support operates outside of the trusted hospital setting.

#### Facilitators in Referring to Peer Support

Nurses also noted facilitators of peer support. Those were mainly related to the personal and affective connections nurses have: ‘*we have had good experiences with [this program] and that is something you communicate to the patients*’ (interview 15, position 125). According to the nurses’ experience, it is important for patients that a first contact is accessible: ‘*what I understand from the African women is that it should definitely not be somebody from their home country. […] You have to imagine that they have not shared their diagnosis with anybody, and this is a giant step; eventually you could be open for a peer of your own cultural background*’ (interview 2, position 55). Another important facilitator was having a peer as a patient: ‘*well, because you have a face and you know them personally, and you can call them if something is playing out or if you think: “wow, they should do something or get in contact with one another”. That is a lot easier*’ (interview 13, position 54).

#### The Effects and Follow-Up of Peer Support

If patients get involved with peer support, the effects, according to the nurses, are overwhelmingly positive. Patients ‘*loose the gravity*’ (interview 8, position 92), ‘*are more open*’ (interview 9, position 95), ‘*regained their self-respect*’ (interview 6, position 64), ‘*have a better understanding and are even more adherent*’ (interview 11, position 95), ‘*step outside of their isolation and shared their diagnosis*’ (interview 16, position 64). For nurses, the follow-up to their patients’ contacts with peers was highly diverse but hardly ever a priority. Some referred patients to peer support but didn’t know whether they actually made contact with the service. Other nurses reported that inquired about the peer support contact based on notes in the electronic patient files or when patients raised the issue themselves. None of the nurses reported that a follow-up system was in place in their treatment centres to facilitate peer support follow-up.

## Discussion

This study describes the experiences of HIV nurses in the Netherlands with peer support. We found that nurses recognise the importance of peer support for their patients and see PLHIV who likely experience a higher social-emotional burden of living with HIV as the main potential target group^24-26.^ Specifically, they mostly propose peer support to women living with HIV who have a migration background. MSM are least referred to peer support as they are thought to have their own support network. This bias could, however, prevent MSM from accessing peer support services they may need since it has been found that the burden of living with HIV remains significant among MSM also.^6,27^ Nurses framed their views and experiences with peer support through the presumed perspective of their patients and the relationship they have with them. They especially identified barriers to linkage to peer support related to the patients’ possible vulnerability and precarity. Also, referrals were experienced as labour intensive, both logistically and at a personal level, and the peer support organisations were perceived as lacking clarity in the services they offers, which might jeopardise the relationship with the nurses. Visibility of and familiarity with patients providing peer support was the most important facilitator identified by participants. The number of PLHIV using peer support remains relatively low, with about 150 people registered in 2022. However, for those patients who do make use of peer support, nurses experience positive effects during their consultations.

Nurses identified patients who they thought experienced the highest social-emotional burden of HIV and the most distance to formal care as the main target group for peer support. This was explicitly linked to the public health concern that PLHIV who are undetectable can no longer transmit the virus and those with elevated viral loads can. Nurses frame peer support as a tool to achieve increased retention in care and adherence to effective treatment^28-30.^ This view of peer support in high-resource setting like the Netherlands is not different from low-resource settings like Sub-Saharan Africa, where the 95-95-95 goals as proposed by UNAIDS as a prerequisite to eliminate HIV transmission are not met. In these settings, (peer) counselling has been described as a social technology aimed at surveillance of patients in order to, ultimately, increasing or ensure adherence^31-35.^ A recent review of the effectiveness of peer support programs for PLHIV found that the most researched benefits of peer support are related to retention in care and viral load suppression, rather than quality of life.^13^ This focus on retention in care as an outcome of peer support is also reflected in the temporal nature of peer support, that is, as an intervention that is needed only until one's HIV status is accepted. Once this prerequisite is achieved can adherence and quality of life be considered within the context of formal care^36-40.^ This emphasis on adherence may trump a focus on improved patient well-being or quality of life. However, participants in our study also indicated that peer support addresses the socio-psychological burden of stigma, isolation, a lack of disclosure which are important indicators of quality of life for PLHIV.^10,22,41,42^ Therefore, beyond biomedical indicators, studies on the impact of peer support should reflect these experiential fields.

The nurses reported doubts and uncertainties about the qualifications of peers, they feared overburdening the peers with the problems of patients or they feared bad experiences might harm their patient. Also, nurses emphasised patients’ potential fear of unwanted disclosure of their HIV status.^43^ The quality of the relationship between patients and nurses and how this informs the provision of formal and informal care remains understudied. However, there is evidence that, in line with our findings, mutual trust is constitutive of effective patient-nurse relations in delivering chronic HIV care.^44,45^ For instance, several studies have suggested that, in order to provide good quality health care, nurses need to cultivate a supportive environment that enables openness and recognises experiences of vulnerability and exclusion.^46^ The importance of the relationship of PLHIV with their nurses in a formal health care context is further illustrated in a study conducted in London which found that people diagnosed since the introduction of effective HIV-treatment prefer to limit disclosure of their diagnosis to the clinical setting since they ‘praised self-reliance and HIV anonymity’ rather than identifying with a community.^43^^(p.1136)^

Nurses also reported that knowing PLHIV who operate as peers is a major facilitator of referring their patients to peer support. The peer's visibility in the clinic is a reminder of the benefits of peer support. The presence of peers removes practical barriers as it facilitates direct access to peer programmes for the nurses. In line with international calls to shift tasks and responsibilities to non-specialist health care providers and community organisation, for instance towards community health teams,^47,48^ our findings indicate that these forms of cooperation between specialist health care providers and other health instances and NGOs requires proximity. A formal connection of peer support providers to the treatment team is crucial both to encourage trust among health care professionals and to lower the threshold for PLHIV to take the step to see a peer.^44,45,49^ This call for better incorporation into formal healthcare by the participants in our study, and the importance of this proximity corresponds with best practices in the UK, where peer support is integrated into formal care. *Positively UK*, a national community organisation which provides peer support, advances education and promotes access to health, has developed national standards for peer support, through cooperation with and endorsement from stakeholders in the formal health sector.^11^ More recently, a user-driven HIV clinic was developed in Norway and identified the incorporation of peer support an important target.^50^ Subsequently, five Norwegian HIV clinics have developed a pilot program inspired by this user-driven clinic and the example of *Positively UK* and found that integrating peer support increased equalising services, enhanced resilience, and modelled positive coping experiences.^51^ The Boston University School for Social Work has coordinated and published a detailed toolkit to support organisations working with peers in order to engage and retain PLHIV in care.^52^ The experience the nurses who participated in our research have with local initiatives that are closely linked to the treatment centre, support this idea of bringing informal care closer to formal care.

This study has potential limitations. Although we managed to recruit nurses from a wide array of treatment centres, we have not elaborated extensively on institutional characteristics of HIV treatment, for example, the nurses’ role within the treatment team. We have also not included the experience of PLHIV or the institutional relationship with the partner organisations that offer peer support which, we suggest, would warrant a study in its own right.

## Conclusions

We set out to study the experiences and perceptions of HIV nurses regarding peer support for PLHIV. Peer support initiatives aim at increasing the quality of life of PLHIV and gain importance in CCM that highlight the importance of patient-centred care and self-care beyond formal care. Our findings indicate that nurses acknowledge the value of peer support but experience significant personal and logistical barriers in making referrals. Nurses evaluate referrals to peer support in the context of their relationship with their patients and consider the public health concerns of losing patients to follow-up. Given the significance of having peers in care and the experiences described for other settings, we suggest that closer integration of peer support into formal care is a possible solution to the existing barriers.

## Supplemental Material

sj-docx-1-jia-10.1177_23259582251372442 - Supplemental material for Perceptions and Experiences of HIV Nurses on Peer Support for People Living With HIV in the Netherlands: A Qualitative Study Supplemental material, sj-docx-1-jia-10.1177_23259582251372442 for Perceptions and Experiences of HIV Nurses on Peer Support for People Living With HIV in the Netherlands: A Qualitative Study by Maarten Bedert, Kevin Moody, John de Wit, Pythia Nieuwkerk and Marc van der Valk in Journal of the International Association of Providers of AIDS Care (JIAPAC)

sj-pdf-2-jia-10.1177_23259582251372442 - Supplemental material for Perceptions and Experiences of HIV Nurses on Peer Support for People Living With HIV in the Netherlands: A Qualitative Study Supplemental material, sj-pdf-2-jia-10.1177_23259582251372442 for Perceptions and Experiences of HIV Nurses on Peer Support for People Living With HIV in the Netherlands: A Qualitative Study by Maarten Bedert, Kevin Moody, John de Wit, Pythia Nieuwkerk and Marc van der Valk in Journal of the International Association of Providers of AIDS Care (JIAPAC)
