# Supplementary material for: Perceptions and Experiences of HIV Nurses on Peer Support for People Living With HIV in the Netherlands: A Qualitative Study
Source: J Int Assoc Provid AIDS Care. 2025 Sep 4;24:23259582251372442. doi: 10.1177/23259582251372442 (PMC12411703; doi:10.1177/23259582251372442)
Supplement: sj-docx-1-jia-10.1177_23259582251372442 - Supplemental material for Perceptions and Experiences of HIV Nurses on Peer Support for People Living With HIV in the Netherlands: A Qualitative Study [file sj-docx-1-jia-10.1177_23259582251372442.docx]

**Interview Guide: Perceptions of Peer Support**

**Project Background**

As a researcher affiliated with Amsterdam UMC, I am currently involved in a project evaluating the impact of peer support on the quality of life of patients. We also want to gain insight into the experiences of healthcare professionals and how they perceive informal care. For this project, we are collaborating with the V&VN. Our goal is to speak with at least one HIV nurse consultant per treatment center.

We would like to be able to properly process the data we collect. Therefore, we would like to record this conversation. The purpose is solely to document our findings accurately. No personal data will be collected, and everything discussed will be processed anonymously. This interview can be terminated at any time.

**Personal and Work History**

- In which HIV treatment center do you work?
- How long have you been working there?
- How large is your team? How many patients do you serve?

**Meaning of Informal Care**

- What does informal care mean to you?
- What does peer support mean to you?
- What are the conditions that peer support must meet?
- Are there themes for which peer support is or is not suitable? Can you give an example?
- Can you describe the type of patient who benefits from peer support?
- Have you noticed an evolution in the role of peer support over the years?
- Where does this evolution come from?
- Has informal care evolved over the years in relation to changing treatments?
- What peer support services are available in the hospital where you work?

(Shiva, HVN, local groups?)

**Role of the Nurse Specialist**

- Can you describe your role as an HIV nurse?
- Have there been situations where you performed other tasks as well?
- Can you describe the boundaries of your responsibilities?

**Experiences with Peer Support / Informal Care**

- Can you describe how the referral to peer support works?
- How do you experience this referral from patients?
- What is it like to convince patients of the value of peer support?
- Would you want to organize things differently regarding peer support?

**Patient Expectations / HIV-Specific Context**

- What types of questions do patients come to you with?
- Do you notice significant differences between patients? Can you elaborate on certain patient types (possibly based on age, years living with HIV, health literacy)?
- Can you explain where your boundaries lie in answering those questions?

**Conclusion**

Thank you very much for participating in this interview. I have covered everything I had prepared. Are there any topics we haven't discussed that you think should be addressed? And do you have any feedback for me?
